# Locomotor kinematics on sand versus vinyl flooring in the sidewinder rattlesnake *Crotalus cerastes*

**DOI:** 10.1242/bio.060146

**Published:** 2023-11-10

**Authors:** Jessica L. Tingle, Brian M. Sherman, Theodore Garland

**Affiliations:** ^1^ Department of Biology, University of Akron, Akron 44325, USA; ^2^ Department of Evolution, Ecology, and Organismal Biology, University of California, Riverside 92521, USA

**Keywords:** Biomechanics, Friction, Granular media, Locomotion, Squamates, Substrate

## Abstract

For terrestrial locomotion of animals and machines, physical characteristics of the substrate can strongly impact kinematics and performance. Snakes are an especially interesting system for studying substrate effects because their gait depends more on the environment than on their speed. We tested sidewinder rattlesnakes (*Crotalus cerastes*) on two surfaces: sand collected from their natural environment and vinyl tile flooring, an artificial surface often used to elicit sidewinding in laboratory settings. Of ten kinematic variables examined, two differed significantly between the substrates: the body's waveform had an average of ∼17% longer wavelength on vinyl flooring (measured in body lengths), and snakes lifted their bodies an average of ∼40% higher on sand (measured in body lengths). Sidewinding may also differ among substrates in ways we did not measure (e.g. ground reaction forces and energetics), leaving open clear directions for future study.

## Teaser

**Summary:** This study provides evidence that sidewinding kinematics differ subtly between a natural and an artificial substrate, sand and vinyl, which have both been used to induce sidewinding in previous studies.

## INTRODUCTION

Terrestrial locomotion necessarily involves physical contact between an organism or machine and the environment. As a result, such substrate characteristics as friction, compliance, rugosity, heterogeneity, and obstacles can impact locomotor kinematics and performance (e.g. Bergmann et al., 2017; Claussen et al., 2002; Clifton et al., 2023; 2020; Collins et al., 2013; Kelley et al., 1997; Li et al., 2010; Redmann et al., 2020). Snakes are an especially interesting system for studying the effects of substrate on terrestrial locomotion because they tend to have a relatively large surface area in contact with the ground, and they can vary the length and location of the contact patch(es); moreover, they differ from limbed tetrapods in that their gait depends more on the environment than on their speed (Gray, 1946; Jayne, 2020).

**Fig. 1.**
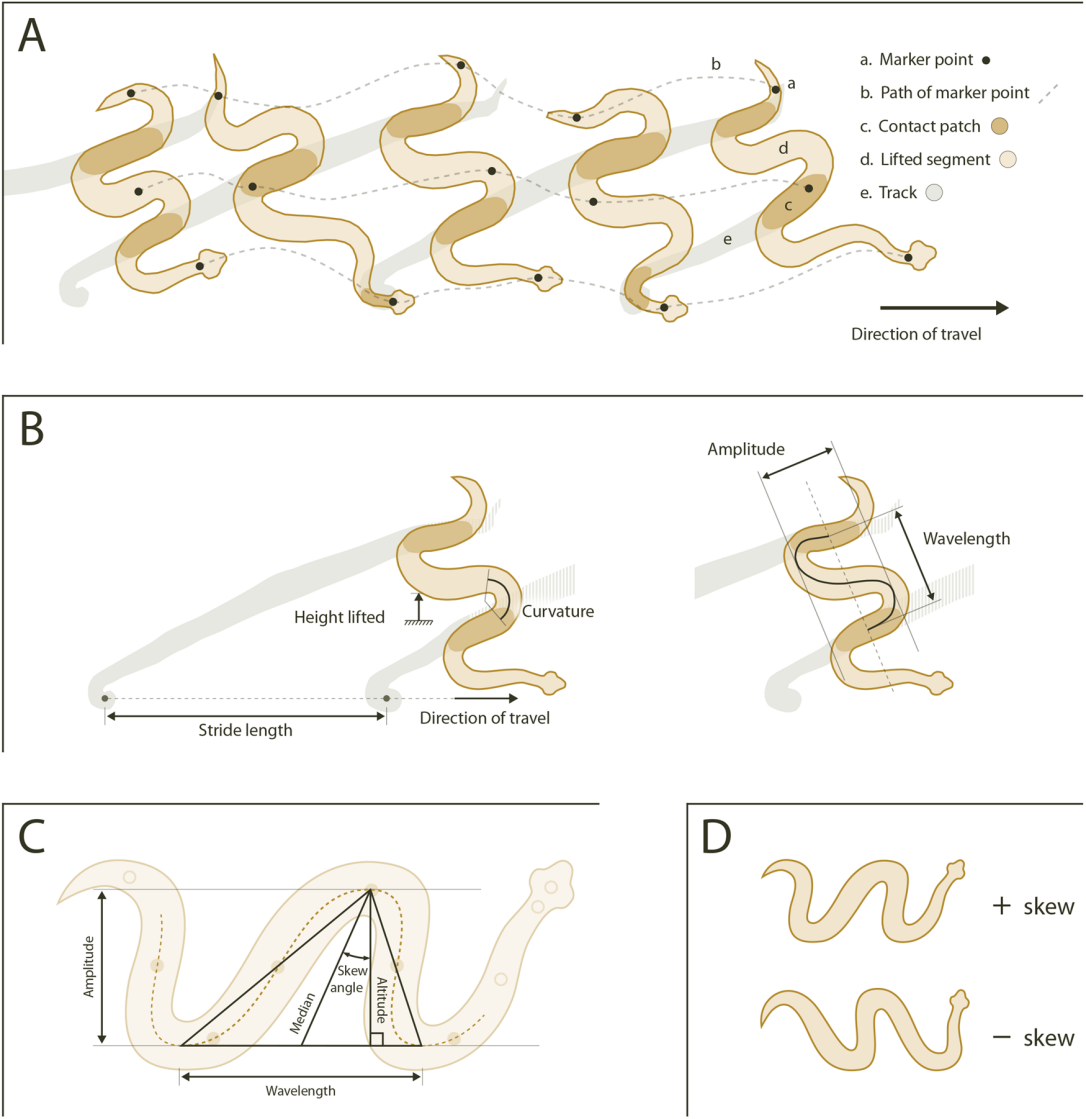
**Sidewinding kinematics.** (A) Sidewinding snakes move in a direction oblique to their body axis, propagating waves that have a horizontal as well as a vertical component. At any given time, some sections of the body remain in static contact with the ground while other sections are lifted up and forward to a new contact patch. (B) The shape of a sidewinder's body can be described using common wave properties, including peak-to-peak amplitude and wavelength. Stride length is the distance between successive tracks in the direction of travel. Because the body axis is oblique to the direction of travel, both amplitude and wavelength contribute to stride length, and their relative contributions are determined by other aspects of the wave's shape, such as skew angle. (C) Wavelength is the distance between successive maxima (crests) or successive minima (troughs). If we draw a triangle between two minima and the maximum in between them (or two maxima and the minimum in between them), then skew angle is the angle between the triangle's median and any line perpendicular to the line connecting the minima (or the maxima). Amplitude is the triangle's altitude, which equals the median times the cosine of the skew angle. (D) Positive skew angle indicates that waves are tilted towards the head, whereas negative skew angle indicates a tail-wards tilt. Figure and caption reproduced from Tingle et al. (2022). Panels A and B are traces from high-speed video of *C. cerastes*, modified with permission from Tingle (2020). Panels C and D are stylized.

Here, we investigate substrate effects on a type of snake locomotion called sidewinding. Sidewinding has a close association with sandy desert environments, probably because it allows snakes to avoid slipping while moving on granular media like sand (Cowles, 1920; Mosauer, 1932; Tingle, 2020). Several viper species have convergently evolved this unusual type of locomotion, which involves holding some parts of the body in static contact with the ground while lifting other parts up and forward to new contact patches farther along the direction of motion (Gans and Mendelssohn, 1971; Gray, 1946; Mosauer, 1930; Mosauer and Wallis, 1928; Tingle, 2020; Tingle and Garland, 2021) (Fig. 1A). Sidewinding vipers differ in their relative use of different types of locomotion, with some employing sidewinding on nearly any surface, and others switching to lateral undulation (the gait most commonly used by snakes and other limbless reptiles) on surfaces like gravel (Gans and Mendelssohn, 1971). Many more species can sidewind facultatively, especially when strongly motivated and/or placed on granular media or smooth surfaces (Tingle, 2020). Additionally, some species also have derived morphological features that are hypothesized to enhance sidewinding, including shorter spinalis muscles (Tingle et al., 2017) and frictionally isotropic microstructure on the ventral scales (Rieser et al., 2021).

Any effect of substrate on sidewinding would be ecologically important because deserts show spatial variability in substrate, including in the size, shape and uniformity of sand grains; the prevalence of dunes (which can have varying morphologies) relative to areas that have been stabilized or semi-stabilized by plants; and the presence of hardpan or other firm surfaces, both natural and human-made, such as paved roads (e.g. Bagnold, 1941; Folk, 1978; Lancaster, 1995; 1989; 1981; Lancaster and Tchakerian, 1996; Sarre and Chancey, 1990). Sidewinding snakes likely encounter many of these different types of substrates, as they can move over large areas and have been observed on different substrate types (Dorfman et al., 2023; Kramer and Schnurrenberger, 1958; Mermod, 1970; Secor, 1994; Subach et al., 2022, 2009). Better understanding of how snakes deal with challenging terrain can also help engineers improve the performance of bio-inspired snake-like robots, which can be used for exploration and search-and-rescue missions in environments where wheeled or limbed robots may struggle to move, including on granular media like sand (Astley, 2022; Gao et al., 2008; Hopkins et al., 2009; Liljebäck et al., 2012).

Substrate effects would also have implications for laboratory studies, which often necessarily use substrates differing from those found in the study species' natural environment (Moore and Clifton, 2023). For studies of sidewinding specifically, researchers have often conducted sidewinding tests on sand that did not come from the animals' natural habitat and/or on smooth laboratory surfaces like linoleum, vinyl flooring, wood, or metal (Brain, 1960; Gasc, 1974; Gray, 1946; Jayne, 1988; Klauber, 1997; Mosauer, 1930; Scanlon, 2001). These studies have never quantified the potential effects of substrate differences on kinematics, and as a result, it is unclear to what degree our interpretation of these studies should be affected by their choice of substrate. It would therefore be very helpful to know which locomotor variables, if any, are affected by these type of substrate differences.

We tested sidewinder rattlesnakes (*Crotalus cerastes* Hallowell 1854) on sand from their natural habitat and on vinyl flooring to determine whether locomotor kinematics are affected. *C. cerastes* is an appropriate study species due to their strong tendency to use sidewinding over other types of locomotion on a wide variety of surfaces (Klauber, 1997; Tingle, 2020) and because we can readily access free-living individuals in the field. We chose sand from their natural habitat and vinyl flooring as our test substrates because, 1) they represent a natural surface and an artificial one that has often been used in laboratory studies, such that the results of this study can help contextualize the ecological relevance of results from laboratory studies and inform the design of future studies, and 2) their very different properties increase the likelihood that we would detect any kinematic differences that might arise from sidewinding on different substrates, as opposed to testing snakes on substrates that differ more subtly from each other. Sand shifts around beneath an animal, behaving somewhat like a solid and somewhat like a fluid (Duran, 2000). Vinyl flooring, on the other hand, is firm but has a low frictional coefficient, such that a snake's skin can easily slide across it. Given that sand and vinyl differ substantially, with one shifting under pressure and the other solid but smooth, we expected that successful locomotion (i.e. forward progress) would require different kinematics on the two surfaces.

## RESULTS

**Fig. 2.**
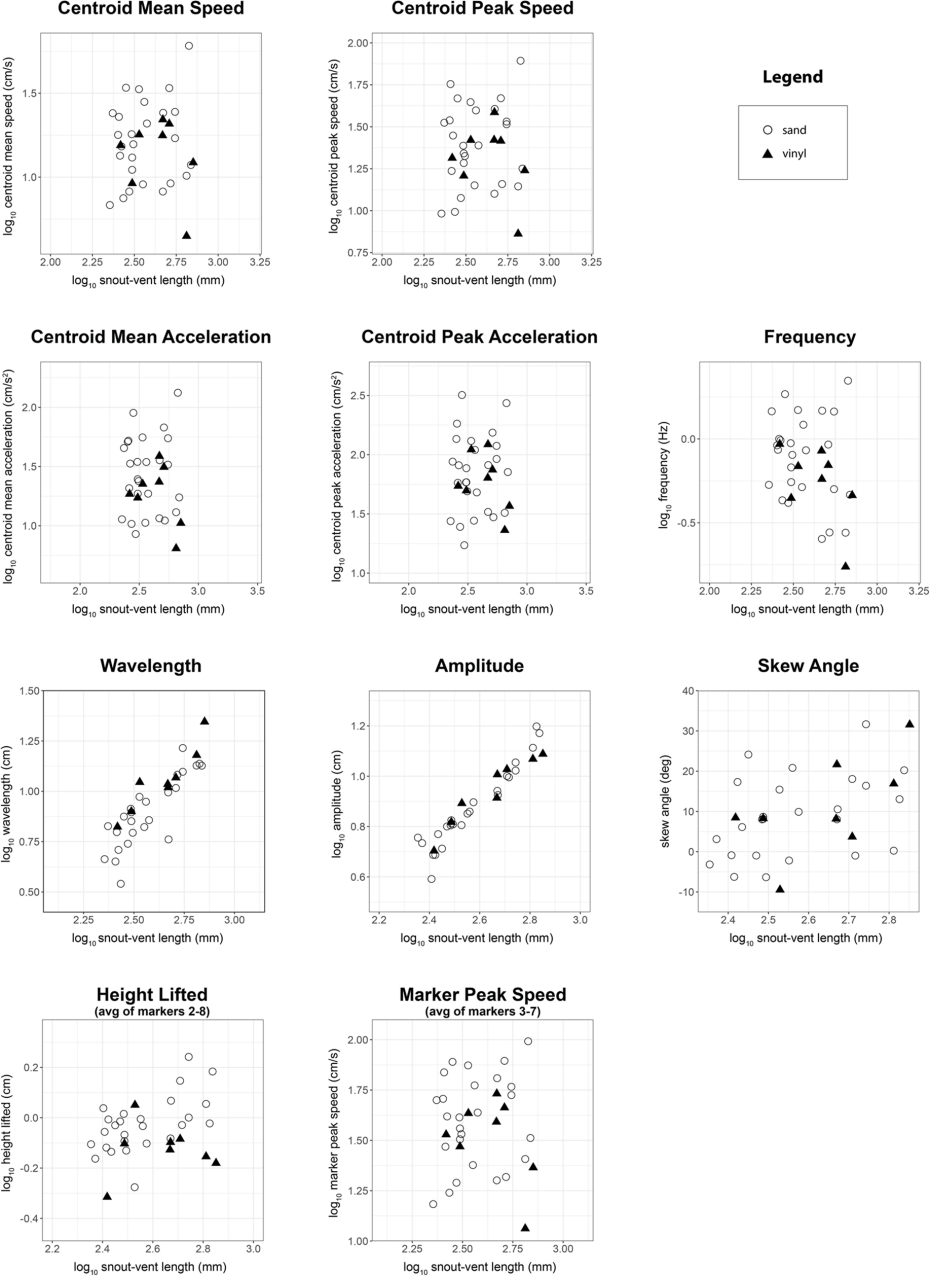
**Comparison of sidewinding kinematics on sand versus vinyl floor tiles with the full sample used in the main analysis.** Scatterplots showing kinematic variables plotted against log_10_ snout-vent length (SVL). Kinematic variables are log_10_ transformed except for skew angle, which cannot be log transformed because it is signed. Open circles represent sand trials (*n*=25 snakes for wavelength, amplitude, and skew angle; *n*=26 for all other variables), whereas filled triangles represent vinyl trials (*n*=8). ANCOVA results indicate that SVL is significantly related to wavelength, amplitude, height lifted, and skew angle (Table 1). Substrate significantly affected wavelength and height lifted (Table 1). Wavelength averaged 0.217 body lengths on sand (range: 0.123-0.297) versus 0.259 body lengths on vinyl flooring (range: 0.223-0.328), a difference of 17.6%. Height lifted averaged 0.027 body lengths on sand (range: 0.014-0.043) versus 0.018 body lengths on vinyl (range: 0.009-0.033), a difference of 40%.

**Fig. 3.**
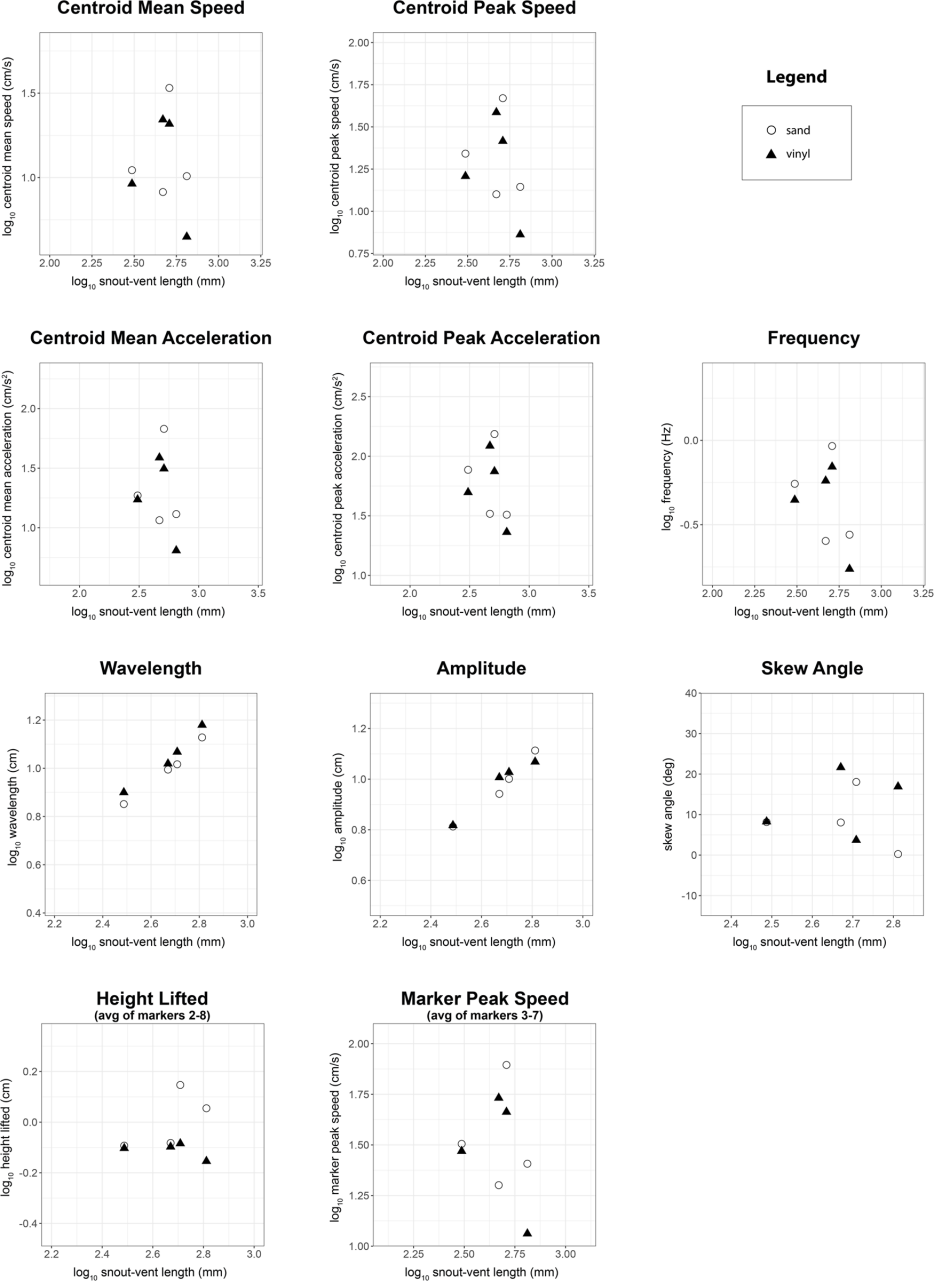
**Comparison of sidewinding kinematics on sand versus vinyl floor tiles, showing only four individuals tested on both substrates.** Scatterplots showing kinematic variables plotted against log_10_ SVL. Kinematic variables are log_10_ transformed except for skew angle, which cannot be log transformed because it is signed. Open circles represent sand trials (*n*=4), whereas filled triangles represent vinyl trials (*n*=4).

Of the ten kinematic variables we examined, with body length as a covariate, two of them differed significantly between substrates: wavelength was longer on vinyl flooring, and sidewinders lifted their bodies higher on sand (Table 1, Fig. 2). Wavelength averaged 0.217 body lengths on sand (range: 0.123-0.297) versus 0.259 body lengths on vinyl (range: 0.223-0.328), a difference of 17.6%. Height lifted averaged 0.027 body lengths on sand (range: 0.014-0.043) versus 0.018 body lengths on vinyl (range: 0.009-0.033), a difference of 40%. Wavelength, amplitude, height lifted, and skew angle increased significantly with snout-vent length (Table 1, Fig. 2). Results from the paired *t*-tests for the four individuals tested on both substrates partially agreed with our main results from the ANCOVA analysis (Fig. 3 presents data for only those four individuals). As for the ANCOVA, wavelength was significantly longer on vinyl (t(3)=6.751, *P*=0.007), but the difference between substrates for height lifted was not significant in the paired *t*-test (t(3)=−1.938, *P*=0.148).

**Table 1.**
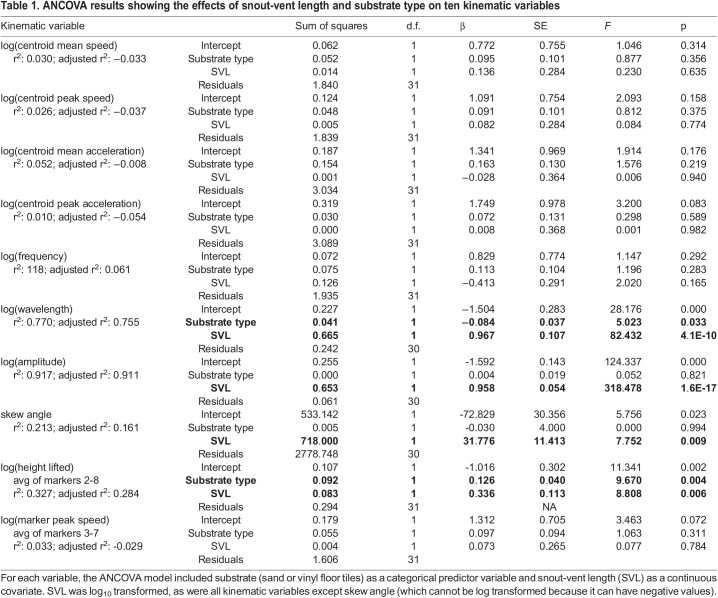
ANCOVA results showing the effects of snout-vent length and substrate type on ten kinematic variables

## DISCUSSION

Previous studies have found kinematic differences on sand versus solid substrates for many kinds of locomotion. For example, eels differ in their kinematics and performance when moving terrestrially on sand versus pebble substrates (Mehta et al., 2020; Redmann et al., 2020), box turtles have shorter strides and lower speeds on sand than on Styrofoam (Claussen et al., 2002), human athletes use different ankle and hip joint kinematics during squat jumps on rigid versus sand surfaces (Giatsis et al., 2004), and a cursorial gecko species was shown to change its body angle and duty factor during transitions from firm to sand surfaces (Naylor and Higham, 2022). On the other hand, one experiment showed that kangaroo rats could hop at 1.8 m s^−2^ on either sand or a solid surface without changing any of the kinematic variables that were measured (hop period, hop length, duty cycle) (Hall et al., 2022). Even within granular media, variation in physical characteristics can affect locomotion. For example, sand particle size may affect running in some lizards (Bergmann et al., 2017), skinks do not burrow as deeply in wet sand as in dry sand (Sharpe et al., 2015), and mudskippers change their locomotor behavior on mud versus dry sand (Naylor and Kawano, 2022).

Our results add not only to the literature on kinematic differences between sand and solid surfaces, but also to a growing body of literature on the ways sidewinding snakes and robots can modulate their kinematics to overcome locomotor challenges. Previous studies have focused on ascending slopes (Hatton and Choset, 2010; Marvi et al., 2014), turning (Astley et al., 2015; Gong et al., 2012), and negotiating obstacles (Astley et al., 2020). As other workers have noted, a more thorough understanding of locomotor control in biological sidewinders can help engineers more effectively coordinate the motion of snake-like robots to achieve a greater range of functions (Astley et al., 2020; 2015; Gong et al., 2016; Marvi et al., 2014).

We provide the first evidence that sidewinding snakes adjust their kinematics at least subtly between substrates. Sidewinders may lift their bodies higher on sand than on vinyl in response to the sand shifting beneath them and/or to clear the ridges of sand that pile up on the edges of their tracks. Speculating why wavelength might be longer on vinyl is more difficult, given that it may change in tandem with other parameters that we were not able to measure.

Given the results of previous studies and the mechanical differences between sand and vinyl flooring, we were surprised to find that only wavelength and height lifted differed, and that they differed relatively subtly, with overlapping distributions in the values for the two substrates (Table 1, Fig. 2). We speculate that morphological adaptations of sidewinder rattlesnakes (*C. cerastes*) might improve the robustness of their locomotion, allowing them to maintain similar kinematics in the face of substrate differences. To give one example of a morphological trait presumed to be an adaptation, *C. cerastes* and some other sidewinding species have a microstructure on the skin of their ventral surface that differs from that of most other snakes (Rieser et al., 2021). This derived microstructure causes their ventral skin to be frictionally isotropic (i.e. to have the same friction coefficient in every direction), which was shown by mathematical modelling to enhance sidewinding performance at the cost of lateral undulation by reducing slipping within the track (Rieser et al., 2021). Species that lack morphological adaptations for sidewinding might face greater challenges controlling their movement, affecting their kinematics. One previous study quantitatively compared sidewinding kinematics in *C. cerastes* and two species that can sidewind facultatively but are not known (or suspected) to have morphological specializations for sidewinding, *Cerberus rynchops* and *Nerodia fasciata* (Jayne, 1986). At times, these two species could be observed to slide within their tracks while otherwise exhibiting an asymmetric movement pattern characteristic of sidewinding (Jayne, 1986). It would be interesting to pursue the question of whether morphological specialization (or lack thereof) affects the magnitude of kinematic change on differing substrates.

Sidewinding locomotion may differ among surfaces in ways we did not quantify. For example, we could not measure slipping, and our method for quantifying the body's waveform could not capture all details of body shape (e.g. local curvatures, the length of regions of static contact with the ground). Perhaps more importantly, we measured only kinematics, and not forces or energetics. The different nature of slipping on sand versus vinyl could have important consequences for ground reaction forces. Additionally, locomotion can be particularly intensive on a shifting surface like sand because not only does the animal have to move its own center of mass relative to the environment, but it also expends energy moving the sand (Lejeune et al., 1998). Therefore, energy expenditure (along with cost of transport) might be expected to differ on shifting versus smooth surfaces. We expect that future studies may also demonstrate differences in ground reaction forces and/or energy use on shifting versus firm substrates. Future studies could also determine whether sand characteristics or other naturally varying aspects of the substrate affect cost of transport, speed, stability, or other biologically relevant performance metrics. Any effects would be consequential for our understanding of free-living animals' habitat use and activity patterns, while informing future experiments on snake locomotion and control of snake-like robots.

## MATERIALS AND METHODS

### Data collection

Our sample consisted of juvenile and adult sidewinder rattlesnakes (*Crotalus cerastes* Hallowell 1854) collected in June and July 2016 at the Barry M. Goldwater Range near Yuma, Arizona, USA. Research procedures were approved by the San Diego State Institutional Animal Care and Use Committee (permit number 16-08-014C), and animals were collected under Arizona State Scientific Collecting Permit SP506470. Within approximately 1 day of capture, we anesthetized the snakes with isoflurane via inhalation, measured snake snout-vent length (SVL) with a measuring tape, and painted ten markers along the body following the protocol described in Tingle et al. (2022). Snake sex and SVL can be found in Supplementary Dataset 1, and additional morphological data collected from the same specimens for a different study (Tingle et al., 2022) can be downloaded from the Dryad data repository (Tingle et al., 2022).

After allowing the snakes to recover from anesthesia for about a day, we conducted locomotor trials on sand and on vinyl flooring. After reducing our sample to trials that did not suffer from video calibration or other issues, the final sample consisted of trials for 26 snakes tested on sand and eight tested on vinyl, with four of those individuals overlapping between the trial types (see below for details on data processing and statistical analysis). The sand trials were previously used for a different study that focused on scaling and relations of morphology with locomotor kinematics (Tingle et al., 2022). The testing arena for sand trials consisted of a shallow 1.2×1.2 m square box containing a 2 cm layer of sand, which was collected on the Barry M. Goldwater Range very near to where the sidewinders were found. We raked and smoothed the sand between trials. The vinyl testing arena consisted of a 1.2×1.2 m area covered in 30.5×30.5 cm squares of 1/8″ standard Excelon vinyl composition tiles (model number 51858), a common flooring in labs (note that such flooring is often colloquially referred to as linoleum, but linoleum is a different material that appears superficially similar). This arena size provided enough space for the snakes, which measured 0.23 to 0.69 m SVL, to complete several sidewinding cycles per trial. Substrate temperatures during testing ranged from 20.4 to 27.2°C, within the range recorded during field observations of active sidewinders (Cowles and Bogert, 1944; Moore, 1978; Signore et al., 2022).

For both trial types, snakes were placed gently into the arena, and if they did not begin moving on their own, they were given the minimum motivation required for them to move, which involved waving snake tongs behind them and/or tapping the tongs on either the substrate or the snake's tail. Trials were recorded with two high-speed cameras (Edgertronic Model SC1; San Jose, CA, USA) positioned approximately 1.5-2 m away from the testing arenas, with one camera on a tall tripod, well above the arena, and the other on a short tripod, close to arena-level. For each individual, we recorded three trials that included at least 2-3 full cycles of sidewinding.

### Data processing

Videos were calibrated and digitized using the MATLAB programs DLTcal5 and DLTdv5 (Hedrick, 2008) to produce 3D coordinates of the ten painted marker points. Our calibration object consisted of several metal rods fixed to each other and to a metal base plate, with markers at regular known intervals. Calibration residuals were <2 pixels. After digitizing, we processed the data and extracted kinematic variables with two custom MATLAB programs, described in detail in our previous paper (Tingle et al., 2022). Briefly, we used the X, Y, and Z coordinates output from DLTdv5 to calculate displacement for all ten markers, which we then smoothed in MATLAB using a 3-pass fourth order Savitzky-Golay filter with a uniform weight distribution. Velocity and acceleration were calculated from smoothed displacement and then smoothed using a single-pass fourth order Savitzky-Golay filter with a uniform weight distribution. After smoothing, we extracted several kinematic variables, including peak speed of individual marker points, mean and peak speed of the centroid of the ten painted markers, mean and peak acceleration of the centroid, frequency of the sidewinding cycles, wavelength and amplitude of the body's waveform, skew angle of the wave, and the height to which the body was lifted (Fig. 1B-D provides diagrams and explanations of kinematic variables).

### Statistical analysis

For each snake, we chose one representative trial based on the following criteria: 1) ruled out trials with obvious issues, like excessively poor video quality or calibration problems, 2) we ruled out trials where the snake did not perform steady-state sidewinding (i.e. trials where the snake stopped or turned); 3) we ruled out trials where our MATLAB program was unable to compute all the variables of interest from the digitizing output, which sometimes occurred if not enough sidewinding cycles were recorded; however, for one individual on sand, all trials lacked some variables, so we chose the trial with the fewest variables lacking; 4) of the remaining trials, we chose the one that captured the greatest number of sidewinding cycles. We originally planned to test for differences in kinematics on vinyl versus sand using a paired design. However, of the ten snakes tested on vinyl, we could not use trials for two of those due to calibration issues, and for four of the eight remaining individuals, their corresponding sand trials were not useable due to calibration issues. Therefore, we compared the eight vinyl trials to the 26 sand trials from our previous study (Tingle et al., 2022). Those sand trials included 22 individuals that were not tested on vinyl, and four individuals that were. For the present study, we used ANCOVAs with type III sums of squares (package *car*; Fox and Weisberg, 2019) to test whether any of the ten kinematic variables differed between the two substrates, with snout-vent length as the covariate. We previously found no significant effect of temperature, sex, or age class on any of the kinematic variables considered (Tingle et al., 2022), so we did not include them as predictors in the current model. As described previously (Tingle et al., 2022), we log_10_ transformed all traits prior to conducting the ANCOVAs except for skew angle, which cannot be log transformed because it is signed. We examined standardized residuals to check for outliers, and we found none using the criterion that outliers would have standardized residuals exceeding ∼3 in magnitude. Residuals were normally distributed, and Levene's test confirmed homogeneity of variance between substrates for all variables.

In addition to our main ANCOVA analysis, we conducted two-tailed paired *t*-tests for the four individuals that were tested on both substrates. Although a sample size of four individuals is at best marginal for such an analysis, we present it for completeness. All statistical analyses were implemented in R 4.2.1 (R Core Team, 2022). Data for trials used in our analysis are provided in Supplementary Dataset 1.

## Supplementary Material

10.1242/biolopen.060146_sup1 Supplementary information

Dataset 1.

## References

[BIO060146C1] Astley, H. C. (2022). Slithering across worlds—snake-inspired robots for extraterrestrial exploration. In *Biomimicry for Aerospace: Technologies and Applications*, (ed. V. Shyam, M. Eggermont and A. F. Hepp), p. 504. Amsterdam: Elsevier.

[BIO060146C2] Astley, H. C., Gong, C., Dai, J., Travers, M., Serrano, M. M., Vela, P. A., Choset, H., Mendelson, J. R., Hu, D. L. and Goldman, D. I. (2015). Modulation of orthogonal body waves enables high maneuverability in sidewinding locomotion. *Proc. Natl. Acad. Sci. USA* 112, 6200-6205. 10.1073/pnas.1418965112 25831489 PMC4434722

[BIO060146C3] Astley, H. C., Rieser, J. M., Kaba, A., Paez, V. M., Tomkinson, I., Mendelson, J. R. and Goldman, D. I. (2020). Side-impact collision: mechanics of obstacle negotiation in sidewinding snakes. *Bioinspir. Biomim.* 15, 065005. 10.1088/1748-3190/abb415 33111708

[BIO060146C4] Bagnold, R. A. (1941). *The Physics of Blown Sand and Desert Dunes*. London: Methuen & Co. Ltd.

[BIO060146C5] Bergmann, P. J., Pettinelli, K. J., Crockett, M. E. and Schaper, E. G. (2017). It's just sand between the toes: how particle size and shape variation affect running performance and kinematics in a generalist lizard. *J. Exp. Biol.* 220, 3706-3716. 10.1242/jeb.161109 29046416

[BIO060146C6] Brain, C. K. (1960). Observations on the locomotion of the south west African adder, *Bitis peringueyi* (Boulenger), with speculations on the origin of sidewinding. *Ann. Transvaal Mus.* 24, 19-24.

[BIO060146C7] Claussen, D. L., Lim, R., Kurz, M. and Wren, K. (2002). Effects of slope, substrate, and temperature on the locomotion of the Ornate Box Turtle, *Terrapene ornata*. *Copeia* 2002, 411-418. 10.1643/0045-8511(2002)002[0411:EOSSAT]2.0.CO;2 15313480

[BIO060146C8] Clifton, G. T., Holway, D. and Gravish, N. (2020). Uneven substrates constrain walking speed in ants through modulation of stride frequency more than stride length. *R. Soc. Open Sci.* 7, 192068. 10.1098/rsos.192068 32269814 PMC7137955

[BIO060146C9] Clifton, G., Stark, A. Y., Li, C. and Gravish, N. (2023). The bumpy road ahead: the role of substrate roughness on animal walking and a proposed comparative metric. *J. Exp. Biol.* 226, jeb245261. 10.1242/jeb.245261 37083141

[BIO060146C10] Collins, C. E., Self, J. D., Anderson, R. A. and McBrayer, L. D. (2013). Rock-dwelling lizards exhibit less sensitivity of sprint speed to increases in substrate rugosity. *Zoology* 116, 151-158. 10.1016/j.zool.2013.01.001 23578935

[BIO060146C11] Cowles, R. B. (1920). A list and some notes on the lizards and snakes represented in the Pomona College Museum. *J. Entomol. Zool. Stud.* 12, 63-66.

[BIO060146C12] Cowles, R. B. and Bogert, C. M. (1944). A preliminary Study of the thermal requirements of desert reptiles. *Bull. Am. Mus. Nat. Hist.* 83, 261-296.

[BIO060146C13] Dorfman, A., Subach, A. and Scharf, I. (2023). Snakes on a slope: strong anti-gravitactic responses and differential habitat use in the Saharan horned viper (*Cerastes cerastes*) in the Negev desert. *R. Soc. Open Sci.* 10, 221652. 10.1098/rsos.221652 36968240 PMC10031405

[BIO060146C14] Duran, J. (2000). *Sands, Powders, and Grains: An Introduction to the Physics of Granular Materials*. New York: Springer-Verlag New York.

[BIO060146C15] Folk, R. L. (1978). Angularity and silica coatings of Simpson Desert sand grains, Northern Territory, Australia. *J. Sediment. Res.* 48, 611-624. 10.1306/212F74E6-2B24-11D7-8648000102C1865D

[BIO060146C16] Fox, J. and Weisberg, S. (2019). *An R Companion to Applied Regression*, 3rd edn. Thousand Oaks, CA: Sage.

[BIO060146C17] Gans, C. and Mendelssohn, H. (1971). Sidewinding and jumping progression of vipers. In *Toxins of Animal and Plant Origin. Gordan and Breach*, (ed. A. De Vries and E. Kochva), pp. 17-38. New York: Science Publishers, Inc.

[BIO060146C18] Gao, J., Gao, X., Zhu, W., Zhu, J. and Wei, B. (2008). Design and research of a new structure rescue snake robot with all body drive system. In *2008 IEEE International Conference on Mechatronics and Automation*, pp. 119-124. IEEE. 10.1109/ICMA.2008.4798737

[BIO060146C19] Gasc, J.-P. (1974). L'interprétation fonctionnelle de l'appareil musculo-squelettique de l'axe vertébral chez les serpents (Reptilia). *Mém. Mus. Natl. D'Hist. Nat. Sér. A Zool.* 83, 1-182.

[BIO060146C20] Giatsis, G., Kollias, I., Panoutsakopoulos, V. and Papaiakovou, G. (2004). Biomechanical differences in elite beach–volleyball players in vertical squat jump on rigid and sand surface. *Sports Biomech.* 3, 145-158. 10.1080/14763140408522835 15079993

[BIO060146C21] Gong, C., Hatton, R. L. and Choset, H. (2012). Conical sidewinding. In *2012 IEEE International Conference on Robotics and Automation*, pp. 4222-4227. IEEE.

[BIO060146C22] Gong, C., Travers, M. J., Astley, H. C., Li, L., Mendelson, J. R., Goldman, D. I. and Choset, H. (2016). Kinematic gait synthesis for snake robots. *Int. J. Robot. Res.* 35, 100-113. 10.1177/0278364915593793

[BIO060146C23] Gray, J. (1946). The mechanism of locomotion in snakes. *J. Exp. Biol.* 23, 101-120. 10.1242/jeb.23.2.101 20281580

[BIO060146C24] Hall, J. K., McGowan, C. P. and Lin, D. C. (2022). Comparison between the kinematics for kangaroo rat hopping on a solid versus sand surface. *R. Soc. Open Sci.* 9, 211491. 10.1098/rsos.211491 35154793 PMC8826122

[BIO060146C25] Hatton, R. L. and Choset, H. (2010). Sidewinding on slopes. In *2010 IEEE International Conference on Robotics and Automation*, pp. 691-696. IEEE.

[BIO060146C26] Hedrick, T. L. (2008). Software techniques for two- and three-dimensional kinematic measurements of biological and biomimetic systems. *Bioinspir. Biomim.* 3, 034001. 10.1088/1748-3182/3/3/034001 18591738

[BIO060146C27] Hopkins, J. K., Spranklin, B. W. and Gupta, S. K. (2009). A survey of snake-inspired robot designs. *Bioinspir. Biomim.* 4, 021001. 10.1088/1748-3182/4/2/021001 19158415

[BIO060146C28] Jayne, B. C. (1986). Kinematics of terrestrial snake locomotion. *Copeia* 1986, 915-927. 10.2307/1445288

[BIO060146C29] Jayne, B. C. (1988). Muscular mechanisms of snake locomotion: an electromyographic study of the sidewinding and concertina modes of *Crotalus cerastes*, *Nerodia fasciata* and *Elaphe obsoleta*. *J. Exp. Biol.* 140, 1-33. 10.1242/jeb.140.1.1 3204332

[BIO060146C30] Jayne, B. C. (2020). What defines different modes of snake locomotion? *Integr. Comp. Biol.* 60, 156-170. 10.1093/icb/icaa017 32271916 PMC7391877

[BIO060146C31] Kelley, K. C., Arnold, S. J. and Gladstone, J. (1997). The effects of substrate and vertebral number on locomotion in the Garter Snake *Thamnophis elegans*. *Funct. Ecol.* 11, 189-198. 10.1046/j.1365-2435.1997.00077.x

[BIO060146C32] Klauber, L. M. (1997). *Rattlesnakes: Their Habits, Life Histories, and Influence on Mankind*, 2nd edn. Berkeley, Los Angeles, London: University of California Press.

[BIO060146C33] Kramer, V. E. and Schnurrenberger, H. (1958). Zur schlangenfauna von Libyen. *Die Aquar. Terr. Zeitschrift* 11, 56-59.

[BIO060146C34] Lancaster, N. (1981). Grain size characteristics of Namib Desert linear dunes. *Sedimentology* 28, 115-122. 10.1111/j.1365-3091.1981.tb01668.x

[BIO060146C35] Lancaster, N. (1989). *The Namib Sand Sea: Dune Forms, Processes and Sediments*. Rotterdam: Balkema.

[BIO060146C36] Lancaster, N. (1995). *Geomorphology of Desert Dunes*. London and New York: Routledge.

[BIO060146C37] Lancaster, N. and Tchakerian, V. P. (1996). Geomorphology and sediments of sand ramps in the Mojave desert. *Geomorphology* 17, 151-165. 10.1016/0169-555X(95)00101-A

[BIO060146C38] Lejeune, T. M., Willems, P. A. and Heglund, N. C. (1998). Mechanics and energetics of human locomotion on sand. *J. Exp. Biol.* 201, 2071-2080. 10.1242/jeb.201.13.2071 9622579

[BIO060146C39] Li, C., Hoover, A. M., Birkmeyer, P., Umbanhowar, P. B., Fearing, R. S. and Goldman, D. I. (2010). Systematic study of the performance of small robots on controlled laboratory substrates. In *Micro- and Nanotechnology Sensors, Systems, and Applications II* (ed. T. George, M. S. Islam and A. K. Dutta). p. 76790Z. SPIE. 10.1117/12.851047

[BIO060146C40] Liljebäck, P., Pettersen, K. Y., Stavdahl, Ø. and Gravdahl, J. T. (2012). A review on modelling, implementation, and control of snake robots. *Robot. Auton. Syst.* 60, 29-40. 10.1016/j.robot.2011.08.010

[BIO060146C41] Marvi, H., Gong, C., Gravish, N., Astley, H., Travers, M., Hatton, R. L., Mendelson, J. R., Choset, H., Hu, D. L. and Goldman, D. I. (2014). Sidewinding with minimal slip: snake and robot ascent of sandy slopes. *Science* 346, 224-229. 10.1126/science.1255718 25301625

[BIO060146C42] Mehta, R. S., Akesson, K., Redmann, E., McCarty–Glenn, M., Ortega, R., Syed, S., Yap–Chiongco, M., Jacquemetton, C. and Ward, A. B. (2020). Terrestrial locomotion in elongate fishes: exploring the roles of morphology and substrate in facilitating locomotion. *J. Zool.* 315, 2-18. 10.1111/jzo.12794

[BIO060146C43] Mermod, C. (1970). Domain vital et déplacements chez *Cerastes vipera* et *Cerastes cerastes* (Reptilia, Viperidae). *Rev. Suisse Zool.* 77, 555-561. 10.5962/bhl.part.75909

[BIO060146C44] Moore, R. G. (1978). Seasonal and daily activity patterns and thermoregulation in the Southwestern Speckled Rattlesnake (*Crotalus mitchelli pyrrhus*) and the Colorado Desert Sidewinder (*Crotalus cerastes laterorepens*). *Copeia* 1978, 439. 10.2307/1443608

[BIO060146C45] Moore, T. Y. and Clifton, G. T. (2023). Jumping over fences: why field- and laboratory-based biomechanical studies can and should learn from each other. *J. Exp. Biol.* 226, jeb245284. 10.1242/jeb.245284 37078872

[BIO060146C46] Mosauer, W. (1930). A note on the sidewinding locomotion of snakes. *Am. Nat.* 64, 179-183. 10.1086/280308

[BIO060146C47] Mosauer, W. (1932). Adaptive convergence in the sand reptiles of the Sahara and of California: a study in structure and behavior. *Copeia* 1932, 72. 10.2307/1435888

[BIO060146C48] Mosauer, W. and Wallis, K. (1928). Beitrage zur kenntnis der reptilienfauana von Tunisien. *Zool. Anz.* 79, 195-207.

[BIO060146C49] Naylor, E. R. and Higham, T. E. (2022). High–speed terrestrial substrate transitions: how a fleeing cursorial day gecko copes with compliance changes that are experienced in nature. *Funct. Ecol.* 36, 471-484. 10.1111/1365-2435.13969

[BIO060146C50] Naylor, E. R. and Kawano, S. M. (2022). Mudskippers modulate their locomotor kinematics when moving on deformable and inclined substrates. *Integr. Comp. Biol.* 62, 1335-1356. 10.1093/icb/icac084 35679069

[BIO060146C51] R Core Team. (2022). R: A Language and Environment for Statistical Computing.

[BIO060146C52] Redmann, E., Sheikh, A., Alqahtani, A., McCarty-Glenn, M., Syed, S., Mehta, R. S. and Ward, A. B. (2020). Terrestrial locomotion in American eels (*Anguilla rostrata*): how substrate and incline affect movement patterns. *Integr. Comp. Biol.* 60, 180-189. 10.1093/icb/icaa016 32251499

[BIO060146C53] Rieser, J. M., Li, T.-D., Tingle, J. L., Goldman, D. I. and Mendelson, J. R. III. (2021). Functional consequences of convergently-evolved microscopic skin features on snake locomotion. *Proc. Natl. Acad. Sci. USA* 118, e2018264118. 10.1073/pnas.2018264118 33547241 PMC8017952

[BIO060146C54] Sarre, R. D. and Chancey, C. C. (1990). Size segregation during aeolian saltation on sand dunes. *Sedimentology* 37, 357-365. 10.1111/j.1365-3091.1990.tb00964.x

[BIO060146C55] Scanlon, J. D. (2001). Sidewinding in terrestrial Australian elapid snakes. *Herpetofauna* 31, 11-18.

[BIO060146C56] Secor, S. M. (1994). Ecological significance of movements and activity range for the Sidewinder, *Crotalus cerastes*. *Copeia* 1994, 631. 10.2307/1447179

[BIO060146C57] Sharpe, S. S., Kuckuk, R. and Goldman, D. I. (2015). Controlled preparation of wet granular media reveals limits to lizard burial ability. *Phys. Biol.* 12, 046009. 10.1088/1478-3975/12/4/046009 26109565

[BIO060146C58] Signore, E., Clark, R. W. and Schraft, H. A. (2022). Temperature-based ambush site selection in sidewinder rattlesnakes (*Crotalus cerastes*). *Southwest. Nat.* 65, 282-287. 10.1894/0038-4909-65.3-4.282

[BIO060146C59] Subach, A., Scharf, I. and Ovadia, O. (2009). Foraging behavior and predation success of the sand viper (*Cerastes vipera*). *Can. J. Zool.* 87, 520-528. 10.1139/Z09-034

[BIO060146C60] Subach, A., Dorfman, A., Avidov, B., Domer, A., Samocha, Y. and Scharf, I. (2022). Foraging behaviour, habitat use and population size of the desert horned viper in the Negev desert. *R. Soc. Open Sci.* 9, 220326. 10.1098/rsos.220326 35774136 PMC9240687

[BIO060146C61] Tingle, J. L. (2020). Facultatively sidewinding snakes and the origins of locomotor specialization. *Integr. Comp. Biol.* 60, 202-214. 10.1093/icb/icaa011 32176289

[BIO060146C62] Tingle, J. L. and Garland, T. Jr. (2021). Morphological evolution in relationship to sidewinding, arboreality and precipitation in snakes of the family Viperidae. *Biol. J. Linn. Soc.* 132, 328-345. 10.1093/biolinnean/blaa208

[BIO060146C63] Tingle, J. L., Gartner, G. E. A., Jayne, B. C. and Garland, T. (2017). Ecological and phylogenetic variability in the spinalis muscle of snakes. *J. Evol. Biol.* 30, 2031-2043. 10.1111/jeb.13173 28857331

[BIO060146C64] Tingle, J. L., Sherman, B. M. and Garland, T. (2022). Scaling and relations of morphology with locomotor kinematics in the sidewinder rattlesnake *Crotalus cerastes*. *J. Exp. Biol.* 225, jeb243817. 10.1242/jeb.243817 35438776 PMC9080748

