# Supplementary material for: Locomotor kinematics on sand versus vinyl flooring in the sidewinder rattlesnake Crotalus cerastes
Source: Biol Open. 2023 Nov 10;12(11):bio060146. doi: 10.1242/bio.060146 (PMC10660788; doi:10.1242/bio.060146)
Supplement: Supplementary information [file biolopen-12-060146-s1.pdf]

Dataset 1.

Available for download at
https://journals.biologists.com/bio/article-lookup/doi/10.1242/bio.060146#supplementary-data
